# Supplementary material for: WordCommentsAnalyzer: A windows software tool for qualitative research
Source: F1000Res. 2018 Sep 4;7:536. Originally published 2018 May 3. [Version 2] doi: 10.12688/f1000research.14819.2 (PMC6137411; doi:10.12688/f1000research.14819.2)
Supplement: Supplementary file 3 [file f1000research-7-17614-s0002.tgz › b2a79496-4ada-47c4-9682-a99ce867836d.zip!/Eight Iranian Health Journals/Salmand_Iranian Journal of Ageing _41.docx]

© Salmand_Iranian Journal of Ageing . This work is licensed under CC BY-NC 4.0 (https://creativecommons.org/licenses/by-nc/4.0/).

Volume & Issue: دوره 3 - شماره 1

Title: بررسی نیازهای سلامتی سالمندان مستمری‌بگیر سازمان تأمین اجتماعی و بازنشستگی کشوری

http://salmandj.uswr.ac.ir/article-1-75-fa.html

اهداف: با پیشرفت علم پزشکی، امید به زندگی افزایش یافته و جوامع را با افزایش جمعیت روبرو ساخته است. برنامه ریزی جهت هرگونه خدمات، نیازمند شناخت نیازهای واقعی سالمندان است. مطالعه حاضر با هدف بررسی نیازهای سلامتی سالمندان سازمان تأمین اجتماعی و سازمان بازنشستگی کشوری طراحی شد. مواد و روش‌ها: این مطالعه از نوع مقطعی می باشد. که در آن 150 سالمند مستمری بگیر (75 نفر در گروه تأمین اجتماعی و 75 نفر در گروه بازنشستگی کشوری) از لحاظ کیفیت زندگی با هم مقایسه شدند. ابزار گردآوری داده‌ها در این مطالعه چک‌لیست مربوط به نیازسنجی و پرسش‌نامه کیفیت زندگی سالمندان لیپاد بود. تجزیه و تحلیل داده‌ها به‌وسیله نرم‌افزار آماری SPSS و با آزمون‌های آماری آنالیز واریانس یک‌طرفه، ضریب همبستگی پیرسون و تی‌تست تجزیه و تحلیل شدند. یافته‌ها: یافته‌ها نشان داد نمره کیفیت زندگی کل سالمندان تأمین اجتماعی بالاتر از بازنشستگی کشوری است (هرچند از نظر آماری معنی دار نیست) و تنها در بعد عملکرد ذهنی‌ و روانی تفاوت معنی‌دار آماری مشاهده شد. نتایج نشان می‌دهد که مهم‌ترین نیاز سالمندان مربوط به درآمد (51/7درصد) آن‌ها می‌باشد. همچنین وضعیت سلامت سالمندان بیانگر آن است که مهم‌ترین مشکلات سالمندان به ترتیب مربوط به مشکلات قلبی‌ و عروقی، عضلانی‌اسکلتی (33/5درصد) و شنوایی و بینایی (19/3درصد) بود. توزیع وسایل کمکی نیز نشان داد که دندان مصنوعی (67/3درصد)، عینک (52درصد) و توالت فرنگی (21/3درصد) بیشترین وسایل مورد استفاده سالمندان است. نتیجه‌گیری: در این مطالعه پایین بودن وضعیت اقتصادی سالمندان نشان می‌دهد که این قشر جامعه نیازمند حمایت‌های بیشتر سازمان‌های اجتماعی و رفاهی می‌باشد. درمجموع اکثر سالمندان مشکلات سلامتی بسیاری را مطرح کردند که توجه برنامه‌ریزان سیستم‌های بهداشتی و اجتماعی را در جهت کمک به سالمندان طلب می‌کند.

Title: بررسی و مقایسه اختلالات روانی سالمندان ساکن در خانه و سالمندان ساکن در سرای سالمندان در شهر تهران

http://salmandj.uswr.ac.ir/article-1-79-fa.html

اهداف: پدیده سالمندی یک مسیر و روند طبیعی می‌باشد. سالمندی در واقع تغییرات بیولوژیکی است که در نحوه زیست ارگانیزم و وضعیت روانی‌اجتماعی فرد در طول زمان ظاهر می‌شود. این تغییرات با کاهش نیروی حیاتی و تطبیقی یعنی کاهش قابلیت انطباق فرد با تغییر شرایط ناگهانی و ناتوانی در ایجاد تعادل مجدد و به تدریج دگرگونی‌هایی را در ساختمان و عمل اعضاء مختلف فرد به وجود می‌آورد و عموماً به افراد بالای 60 سال اطلاق می شود. پژوهش حاضر براین بوده است که تبعات روانی و عاطفی جدایی سالمندان از خانه و خانواده را مورد بررسی قرار دهد. مواد و روش‌ها: این تحقیق، یک مطالعه مقایسه‌­ای بین سالمندان ساکن در خانه و سالمندان ساکن در سرای سالمندان ( کهریزک تهران) می­باشد بدین منظور، از بین کلیه سالمندان ساکن در سرای سالمندان کهریزک و سالمندان ساکن در خانه (در دسترس)، تعداد 50 سالمند ساکن در سرای سالمند (25 زن و 25 مرد) و 50 سالمند ساکن در خانه (25 زن و 25 مرد) مجموعاً 100 نفر از سالمندان بالای 65 سال از کل جامعه هدف در قالب نمونه آماری انتخاب گردید. ابزار این پژوهش، پرسش‌نامه اختلالات عصبی، فکری و عاطفی SCL 90 می‌باشد. فرضیه این پژوهش، مقایسه اختلالات روانی سالمندان ساکن در خانه با سالمندان ساکن در سرای سالمندان است. یافته‌­ها: با توجه به تحلیل‌های آماری انجام شده، نشان داده شد که بین وضعیت عاطفی و روانی سالمندان ساکن در خانه و سالمندان ساکن در سرای سالمندان در هر دو گروه زن و مرد در سطوح الفای %5 و %1 تفاوت معنی‌داری وجود دارد. نتیجه­‌گیری: با توجه به یافته‌­های این مطالعه استنباط می‌­شود که سالمندان ساکن در سرای سالمندان از وضعیت روانی و عاطفی نامطلوب‌تری نسبت به سالمندان ساکن در خانه برخوردارند.

Title: اثرات فشار خون بالا بر کیفیت زندگی سالمندان عضو فرهنگسرای سالمند شهر تهران

http://salmandj.uswr.ac.ir/article-1-80-fa.html

اهداف: امروزه با توجه به عوامل گوناگون جمعیت سالمند بسرعت رو به رشد می‌باشند و از طرفی کیفیت زندگی در سالمندان تحت تأثیر عوامل مختلف قرار دارد افزایش فشار خون نیز در سالمندان بسیار شایع می باشد. در این پژوهش به بررسی اثرات پر فشاری خون بر کیفیت زندگی سالمندان پرداخته شده است. مواد و روش‌ها: هدف از این تحقیق مقطعی، توصیفی و تحلیلی بررسی کیفیت زندگی سالمندان مبتلا به فشار خون بالا در شهر تهران است. جامعه پژوهش، سالمندان عضو فرهنگسرای سالمند بود که 300 نفر نمونه، بصورت تصادفی از افراد 60 سال و بالاتر انتخاب شدند که 191 نفر سالم و بدون شکایت از بیماری، 23 نفر مبتلا به فشار خون بالا و سایر شرکت‌کنندگان از بیماری‌های دیگر در رنج بودند. اطلاعات از طریق پرسش‌نامه استاندارد WHOQOL-BREF با چهار حیطه جسمانی، روانی، اجتماعی و محیطی و باروش مصاحبه رودر رو، جمع‌آوری و جهت بررسی ابتدا به Excel وارد و سپس به منظور تجزیه و تحلیل اطلاعات حاصله، از Anova و ضریب همبستگی اسپیرمن در SPSS استفاده گردید. یافته‌ها: بر اساس یافته‌ها، 223 نفر زن (%74/33) و 77 نفر مرد (%25/66) با دامنه سنی 85-60 و میانگین سنی 68/08 سال در مطالعه شرکت داشتند. نتایج پژوهش حاکی از این بود که بین حیطه‌های (domains) جسمانی، روانی و محیطی کیفیت زندگی و کیفیت زندگی به‌طور کلی، در افراد سالم و فشار خون بالا، تفاوت معنی‌داری مشاهده گردید (P<0/000) درحالی‌که در حیطه اجتماعی تفاوت معنی‌دار مشاهده نگردید. درحیطه جسمانی در مقایسه بین دو گروه، ارتباط بین تحصیلات و کیفیت زندگی بسیار قوی و معنی دار بوده است (r=0/256 و P<0/001). در مقایسه کیفیت زندگی با سن (P<0/03) و با وضعیت تأهل ( P<0/039) و با جنس (P<0/03) نیز تفاوت معنی‌داری مشاهده گردید. نتیجه‌گیری: یافته‌های فوق نشان داد که فشار خون بالا در سالمندان درحیطه‌های جسمانی، روانی و محیطی و همچنین به‌طور کلی موجب کاهش و نزول کیفیت زندگی می‌گردد. بنا بر این ممکن است با اقدامات پیشگیرانه و یا درمان به هنگام این عارضه سطح کیفیت زندگی سالمندان را ارتقاء داد.

Title: کیفیت زندگی در زنان سالمند عضو کانون روزانه جهان‌دیدگان شهر شیراز 1386

http://salmandj.uswr.ac.ir/article-1-83-fa.html

اهداف: پیر شدن یک تجربه شخصی و اجتماعی است. این تجربیات می تواند برای عده ای خوشحال کننده و برای عده‌ای دیگر غم‌انگیز و ملالت بار باشد. جهت تصمیم‌گیری و برنامه‌ریزی مشکلات این گروه، درنتیجه بهبود کیفیت زندگی سالمندان باید شناخت دقیقی از مشکلات آن‌ها داشته باشیم. مواد و روش‌ها: این مطالعه به روش مقطعی و توصیفی‌تحلیلی در کانون جهاندیدگان شهر شیراز در سال 1386 انجام شده است. جامعه مورد پژوهش در این تحقیق 400 زن سالمند 79–60 سال بود که از این تعداد 232 نفر به صورت تصادفی انتخاب شدند. جهت جمع‌آوری داده‌ها از پرسش‌نامه کیفیت زندگی لیپاد با دو بخش کیفیت زندگی اصلی و کیفیت زندگی تسهیل کننده استفاده شد. داده‌ها با آزمون‌های آنالیز واریانس یک‌طرفه، تی تست و ضریب همبستگی پیرسون در نرم افزار spss مورد تجزیه‌و‌تحلیل قرار گرفت. یافته‌ها: یافته ها نشان دهنده وجود اختلاف معنی دار بین میانگین کیفیت زندگی (در هر دو بخش و در کل) با وضعیت تاهل می‌باشد (P<0/05) به‌طوری که کیفیت زندگی زنان سالمند در کل در افراد ازدواج نکرده از وضعیت بهتری نسبت به سایرین برخوردار بود و کیفیت زندگی در سالمندان متارکه کرده یا طلاق گرفته و بیوه از کمترین سطح برخوردار بود. بین هر دو بخش کیفیت زندگی همچنین کیفیت زندگی درکل با سطح تحصیلات رابطه وجود داشت هرچند تنها در بخش کیفیت زندگی اصلی رابطه معنی‌داری مشاهده گردید (P=0/047). بین کیفیت زندگی (کیفیت زندگی اصلی و در کل) با سن زنان سالمند رابطه معنی‌داری یافت شد (P<0/05) به طوری که با افزایش سن میانگین کیفیت زندگی کاهش می یافت. همچنین بین کیفیت زندگی (هر دو بخش کیفیت زندگی و در کل) با میزان درآمد زنان سالمند رابطه معنی داری و مسقیم یافت شد (P<0/05). بین میانگین کیفیت زندگی (در هر دو بعد و در کل) و ابتلا به بیماری‌های مزمن نیز تفاوت معنی‌داری مشاهده گردید (P<0/05). نتیجه‌گیری: یافته‌های حاصل از این پژوهش بیانگر تاثیر برخی فاکتورها مانند بیماری‌ها، سطح تحصیلات وضعیت تأهل، سن، درآمد و شغل بر کیفیت زندگی زنان سالمند می‌باشد بنابراین استراتژِی‌ها باید در جهت متغیرهای مذکور برای طراحی و حل مشکلات فعلی و آینده سالمندان هدایت گردد.

Title: سالمندآزاری در شهر اهواز و ارتباط آن با برخی مشخصه‌های فردی و اجتماعی

http://salmandj.uswr.ac.ir/article-1-84-fa.html

اهداف: سالمندان از جمله گروه‌های آسیب‌پذیر در جامعه می‌باشند و پدیده سالمندآزاری از جمله چالش‌های مطرح درحیطه مراقبت و حمایت از سالمندان است. مطالعه کنونی به منظور تعیین شیوع سوءرفتار با سالمندان و ارتباط آن با برخی از مشخصه‌های فردی و اجتماعی انجام شده است. مواد و روش‌ها: مطالعه حاضر مقطعی بوده که به شکل نمونه‌گیری تصادفی در روی 385 سالمند بالاتر از 60 سال ساکن شهر اهواز در سال 1385 صورت گرفته است. از پرسش‌نامه بررسی سوءرفتار با سالمند با روایی محتوا و پایایی %87 جهت جمع آوری اطلاعات استفاده شد و و از آمار توصیفی و تحلیلی جهت تجزیه و تحلیل اطلاعات استفاده شد. یافته‌ها: میانگین سنی سالمندان 70 سال بوده است، شیوع سوءرفتار جسمی %5/10، روحی %9/16، غفلت %7/31، طرد %12، و مالی %25 بوده است. بین وجود بیماری (P=0/009)، نوع زندگی و وابستگی جسمی به دیگران (P=0/001) با سوء رفتار ارتباط معنی‌داری دیده شد. نتیجه‌گیری: علی‌رغم تأکید زیادی که در فرهنگ ما برای احترام به سالمند وجود دارد، سالمندان انواع مختلف سوءرفتار خصوصاٌ مالی و غفلت را بیشتر تجربه می‌کنند لذا شناخت دقیق سوءرفتار با سالمندان در جامعه می‌تواند آگاهی و حساسیت افراد و سازمان‌های مرتبط با این پدیده را بالا برده تا در جهت پیشگیری و طراحی سیستم شناسایی سالمندان در معرض خطر اقدامات موثری را انجام دهند.

Title: مقایسه میزان تأثیر درد بر ابعاد مختلف زندگی سالمندان و افراد مبتلا به درد مزمن

http://salmandj.uswr.ac.ir/article-1-85-fa.html

اهداف: هدف از انجام این مطالعه بررسی تأثیر درد بر ابعاد مختلف زندگی سالمندان مبتلا به درد مزمن بود. مواد و روش‌ها: 585 نفر از افراد مبتلا به درد مزمن در ناحیه پاها، کمر، دستها، گردن و شانه در این پژوهش شرکت داشتند. این افراد شامل دو گروه بودند: 1) افراد سالمند در سن 60 سالگی و یا بالاتر (77=n)؛  2) افراد در سنین کمتر از 60 سالگی (508=n). ابزاری که به منظور بررسی ابعاد چندگانه درد مزمن تهیه شده پرسشنامه چند بعدی درد وست‌هاون –ییل WHYMPI است. یافته‌ها: دو گروه در سه مقیاس از ابعاد مهم تجربه درد تفاوت معنی‌داری با هم دارند. این سه مقیاس عبارتند از: تداخل درد در زندگی روزانه، حمایت و وابستگی به همسر یا فرد مهم زندگی و شدت درد. افراد سالمند به‌طور معنی‌داری درد شدیدتری را تجربه می‌کنند و این موضوع باعث تداخل بیشتر درد در زندگی روزانه و حمایت و وابستگی به همسر یا فرد مهم زندگی آنان می‌شود. و در نهایت نتایج نشان داد دو گروه در سه مقیاس از مقایسه‌ای ارزیابی و گزارش بیمار از فعالیت‌های متداول و معمولی زندگی تفاوت معنی‌داری با هم داشتند. این سه مقیاس عبارت‌اند از: فعالیت‌های دور از خانه، فعالیت‌های اجتماعی و فعالیت‌های عمومی. نتیجه‌گیری: شدت درد بیشتر در گروه سالمندان موجب تداخل بیشتر درد در زندگی روزانه و میزان حمایت و وابستگی به همسر یا فرد مهم زندگی آنان می‌شود. از طرف دیگر به نظر می رسد درد مزمن فعالیت‌های دور از خانه، فعالیت‌های اجتماعی و فعالیت‌های عمومی سالمندان مبتلا به درد مزمن را بیشتر از بیماران عادی مبتلا به درد مزمن تحت تأثیر قرار می‌دهد. لذا در مراقبت از سالمندان مبتلا به درد مزمن باید بیشتر از افراد و بیماران عادی مبتلا به درد مزمن به فکر فعالیت‌های عمومی، اجتماعی و بیرون خانه آن‌ها بود.

Title: تأثیر مشاوره گروهی با رویکرد معنادرمانی بر سطح سلامت روان زنان سالمند

http://salmandj.uswr.ac.ir/article-1-86-fa.html

اهداف: پیری بیماری نیست، بلکه واقعیتی است اجتناب ناپذیر در گذرگاه طبیعی رشد. با این که برخی از اختلالات روانی، از جمله اضطراب و افسردگی، در سالمندی شایعاند در بسیاری از موارد نادیده گرفته شده یا به عنوان جزئی از فرایند سالمندی کم اهمیت شمرده می شوند. درمان‌های دارویی و مشاوره فردی و گروهی می‌توانند در رهایی سالمند از تعارضات عاطفی و فشارهای روانی سودمند باشند. با توجه به دشواری و پیچیدگی درمان دارویی در سالمندان، کاربرد روش‌های غیردارویی به تنهایی یا در صورت ضرورت به شکل ترکیب با درمان دارویی توصیه شده است.این پژوهش با هدف بررسی تاثیر مشاوره گروهی بارویکرد معنا درمانی بر سطح سلامت روان زنان سالمند صورت پذیرفته است. مواد و روش‌ها: برای انجام این پژوهش از طرح نیمه تجربی پیش آزمون–پس آزمون با گروه گواه استفاده شده است. از میان 160 بانوی سالمند 65 سال و بالاتر ساکن در آسایشگاه کهریزک تهران نمونه‌ای با حجم 18 نفر به‌طور تصادفی انتخاب شد. سپس آزمودنی‌ها بر مبنای نمرات سلامت روان به‌دست‌آمده از اجرای پرسش‌نامه سلامت عمومی GHQ-28 (پیش آزمون) همتاسازی و در دو گروه آزمایش و گواه (هر گروه 9نفر) جایگزین شدند. 10جلسه مشاوره گروهی معنادرمانی به صورت هفتگی برای گروه آزمایش اجرا گردیده در پایان پرسش‌نامه سلامت عمومی GHQ-28 (پس آزمون) مجددا برای هر دو گروه اجرا شد. داده‌های به‌دست‌آمده از اجرای پیش‌آزمون و پس‌آزمون به کمک آمار توصیفی و آمار استنباطی (آزمون T برای گروه‌های وابسته) مورد تجزیه‌و‌تحلیل قرار گرفت. یافته‌ها: نتایج به دست آمده از این تجزیه و تحلیل حاکی از آن بود که کاربرد مشاوره گروهی بارویکرد معنا درمانی موجب افزایش سطح سلامت روان، کاهش میزان اضطراب و فشار روانی (P=0/015) و نیز کاهش اختلال در کنش اجتماعی زنان سالمند گردیده است (P= 0/005). اما در کاهش میزان شکایات جسمانی (P=0/13) و افسردگی (P=0/86) آن‌ها تأثیر چشمگیری نداشته است. نتیجه‌گیری: کاربرد گروه‌درمانی با رویکرد معنادرمانی می‌تواند در ارتقای سطح سلامت روان زنان سالمند ساکن آسایشگاه‌ها اثربخش باشد.

Title: خدمات مراقبتی سالمندی در ایالات متحده آمریکا با نگاهی اجمالی به ایران

http://salmandj.uswr.ac.ir/article-1-87-fa.html

اهداف: تدوین نظام جامع خدمات ویژه جمعیت سالمندی امری ضروری به نظر می‌رسد. در این راستا استفاده از تجارب کشورهای پیشرفته در این حوزه بالحاظ نمودن ویژگی‌های فرهنگی می‌تواند به کشورهای درحال توسعه کمک نماید. هدف از مطالعه حاضر بررسی نظام خدمات جامع سالمندی در آمریکا با نگاهی اجمالی به این خدمات در ایران می‌باشد. مواد و روش‌ها: این مقاله یک مطالعه اسنادی است که به بررسی متون در حوزه نظام مراقبت جامع سالمندی در ایران و آمریکا می­‌پردازد. جامعه پژوهش مجموعه مکتوبات و مستندات مرتبط با موضوع مطالعه می­‌باشد و نمونه‌گیری به صورت هدفمند بر اساس میزان وابستگی، ‌ارتباط به مضامین تحت مطالعه و دربرگیرنده کلیدواژه‌­های مرتبط بوده است. یافته‌ها: در این مطالعه ضمن تشریح خصوصیات جمعیتی اجتماعی سالمندان ایران و آمریکا، وضعیت سلامت سالمندان، ابعاد نظام تأمین اجتماعی و ارائه خدمات بهداشتی درمانی مقایسه شده است. بارزترین یافته در مقایسه این دو سیستم وجود نظام تأمین اجتماعی قوی و متنوع در آمریکاست که تحت عنوان «مدی کر» و «مدی کید» مطرح می‌باشد. تاکید بر خدمات درازمدت، پوشش خدمات کوتاه‌مدت و تنوع در سیستم ارائه خدمات مراقبتی بر جامعه در نظام آمریکا بارز می‌باشد. نتیجه‌گیری: تأمین اجتماعی یک منبع درآمد مهم برای بازنشستگان و سالمندان در آمریکا و سایر کشورهای توسعه یافته به حساب می‌آید. بدون این منبع درآمدی دو سوم سالمندان در فقر خواهند بود. در ایران نیز تأمین اجتماعی و بیمه‌های سالمندان باید برای افزایش کیفیت زندگی سالمندان تقویت شود. در ایران سازمان‌های مردم‌نهاد مرتبط با حوزه سلامت و رفاه سالمندان می­‌تواند پاسخگوی مناسبی برای رفع مشکلات این قشر باشد.
